# Combination of a Fast Cleanup Procedure and a DR-CALUX^®^ Bioassay for Dioxin Surveillance in Taiwanese Soils

**DOI:** 10.3390/ijerph110504886

**Published:** 2014-05-06

**Authors:** Ding-Yan Lin, Yi-Pin Lee, Chiu-Ping Li, Kai-Hsien Chi, Bo-Wei P. Liang, Wen-Yao Liu, Chih-Cheng Wang, Susana Lin, Ting-Chien Chen, Kuei-Jyum C. Yeh, Ping-Chi Hsu, Yi-Chyun Hsu, How-Ran Chao, Tsui-Chun Tsou

**Affiliations:** 1 Emerging Compounds Research Center, Department of Environmental Science and Engineering, National Pingtung University of Science and Technology, Neipu, Pingtung County 912, Taiwan; E-Mails: p9831003@mail.npust.edu.tw (D.-Y.L.); chen5637@mail.npust.edu.tw (T.-C.C.); kjyeh@mail.npust.edu.tw (K.-J.C.Y.); 2 Division 5 of the Environmental Analysis Laboratory, Environmental Protection Administration, Zhongli, Taoyuan County 320, Taiwan; E-Mails: yplee@mail.niea.gov.tw (Y.-P.L.); cplee@mail.niea.gov.tw (C.-P.L.); 3 Institute of Environmental and Occupational Health Science, National Yang-Ming University, Beitou Dist., Taipei City 112, Taiwan; E-Mail: khchi2@ym.edu.tw; 4 Environmental Engineering Department Ⅱ, Sinotech Engineering Consultants, Ltd., Neihu Dist., Taipei City 114, Taiwan; E-Mail: powerl@mail.sinotech.com.tw; 5 MWH Americas Inc., Taiwan Branch, Daan Dist., Taipei City 106, Taiwan; E-Mails: Adam.W.Liu@mwhglobal.com (W.-Y.L.); 174600@cpdce.com.tw (C.-C.W.); 6 Department of Bioenvironmental Systems Engineering, National Taiwan University, Daan Dist., Taipei City 106, Taiwan; 7 International College, National Pingtung University of Science and Technology, Neipu, Pingtung County 912, Taiwan; E-Mail: susanalin@mail.npust.edu.tw; 8 Department of Safety, Health and Environmental Engineering, National Kaohsiung First University of Science and Technology, Yanchao Dist., Kaohsiung City 811, Taiwan; E-Mail: pchsu@nkfust.edu.tw; 9 Department of Environmental Engineering, Kun Shan University, Yongkang Dist., Tainan City 710, Taiwan; E-Mail: ychsu22@yahoo.com.tw; 10 Division of Environmental Health and Occupational Medicine, National Health Research Institutes, Zhunan, Miaoli County 350, Taiwan; E-Mail: tctsou@nhri.org.tw

**Keywords:** polychlorinated dibenzo-*p*-dioxins/furans, soil, aryl hydrocarbon receptor (AhR), bioassay, DR-CALUX^®^, dioxin survey

## Abstract

Our goal was to determine dioxin levels in 800 soil samples collected from Taiwan. An *in vitro* DR-CALUX^®^ assay was carried out with the help of an automated Soxhlet system and fast cleanup column. The mean dioxin level of 800 soil samples was 36.0 pg-bioanalytical equivalents (BEQs)/g dry weight (d.w.). Soil dioxin-BEQs were higher in northern Taiwan (61.8 pg-BEQ/g d.w.) than in central, southern, and eastern Taiwan (22.2, 24.9, and 7.80 pg-BEQ/g d.w., respectively). Analysis of multiple linear regression models identified four major predictors of dioxin-BEQs including soil sampling location (*β* = 0.097, *p* < 0.001), land use (*β* = 0.065, *p* < 0.001), soil brightness (*β* = 0.170, *p* < 0.001), and soil moisture (*β* = 0.051, *p* = 0.020), with adjusted R^2^ = 0.947 (*p* < 0.001) (n = 662). An univariate logistic regression analysis with the cut-off point of 33.4 pg-BEQ/g d.w. showed significant odds ratios (ORs) for soil sampling location (OR = 2.43, *p* < 0.001), land use (OR = 1.47, *p* < 0.001), and soil brightness (OR = 2.83, *p* = 0.009). In conclusion, four variables, including soil sampling location, land use, soil brightness, and soil moisture, may be related to soil-dioxin contamination. Soil samples collected in northern Taiwan, and especially in Bade City, soils near industrial areas, and soils with darker color may contain higher dioxin-BEQ levels.

## 1. Introduction

Polychlorinated dibenzo-*p*-dioxins/furans (PCDD/Fs) are halogenated aromatic hydrocarbons and recognized as environmental endocrine disruptors. There has recently been an increased interest in environmental monitoring of PCDD/Fs, owing to their potentially hazardous effects on ecosystems and human health, as well as the risks associated with PCDD/Fs bioaccumulation and biomagnification in the food chain [1,2]. PCDD/Fs are persistent, ubiquitous contaminants that are released into the environment as unwanted byproducts of incomplete combustion or impurities in various chemicals [3,4,5]. Because of their chemical stability, lipid solubility, and resistance to chemical and physical degradation, PCDD/Fs can move away from their source regions to more remote locations through atmospheric long-range transport, thus accumulating in the atmosphere over the land and ocean [6,7], in soils [8,9,10], sediments [11,12], terrestrial and aquatic animals [13,14], and humans [15,16,17].

Soil can be significantly contaminated with PCDD/Fs through airborne transport, and it is a potential route for human or animal exposure to PCDD/Fs [18,19]. Many environmental scientists have examined soils near stationary points with high dioxin emissions [10,13,20,21]. Recently, several studies have carried out soil dioxin surveys in various local regions [22,23,24], but only a few studies have used bioassays to determine soil dioxin levels [8,25,26,27,28,29]. A high correlation has been found between dioxin levels measured using the *in vitro* aryl-hydrocarbon-receptor (AhR) reporter gene assay and dioxin levels measured using chemical assays [8,30,31]. Although it is a fast-screening and semi-quantitative method of measuring dioxin levels in soil, the AhR reporter gene assay has been used in only a few large-scale soil-contamination studies or large-scale surveillances of dioxin-contaminated soil [27,29].

AhR reporter gene assays, including the chemically activated luciferase gene expression (CALUX) assay, have been widely used to measure dioxin levels in different environmental matrices, including soils [27,32] and biological samples, such as fish [33] and breast milk [34]. As compared with chemical assay approaches of large-scale dioxin surveillance, e.g., high-resolution gas chromatography/ high-resolution mass spectrometry (HRGC/HRMS), the pros and cons of the DR-CALUX^®^ bioassay were listed in the following two sentences. For the advantages, the DR-CALUX^®^ assay is rapid, low-cost, fast-screening, and semi-quantitative [35]. DR-CALUX^®^ bioassay has several disadvantages including false-positive and false-negative outcomes, requirement of extensive cleanup, easy interference by the non-AhR ligand, for examples: As^2+^, Cd^2+^, and arecoline, and performance of total dioxin-BEQs without presenting the congener-specific dioxin patterns [16,36,37,38]. A multicolumn cleanup procedure (with the following sequence of cleanup columns: silica, alumina, and carbon columns) followed by HRGC/HRMS is the widely-used global standard method for PCDD/Fs analysis. However, both HRGC/HRMS methods and AhR reporter gene assays require extensive cleanup processes after extraction to remove substances that can seriously affect the performance of analytical outcomes. In our previous studies [16,36,37], several non-AhR-ligand chemicals, such as arsenite (As^3+^), arecoline, and cadmium (II) (Cd^2+^), were shown to have the potential to interfere with 2,3,7,8-tetrachlorodibenzo-*p*-dioxin (TCDD)-induced dioxin response element (DRE)-driven AhR luciferase expression, indicating that extensive cleanup procedures are critical for dioxin detection using the AhR reporter gene assays, including the DR-CALUX^®^ assay.

In the present study, a national surveillance of dioxin-contaminated soil was carried out in Taiwan. To improve the efficiency of PCDD/F measurements in environmental samples, an automated Soxhlet system was combined with an effective cleanup system to clean up and extract dioxin from 800 soil samples prior to DR-CALUX^®^ assay for determining their dioxin levels. A Soxtherm extractor combined with a CAPE-coupled activated carbon-acid silica column set (CAPE column; CAPE Technologies, South Portland, ME, USA) used by the Environmental Analysis Laboratory (EAL) of the Environmental Protection Administration (EPA) in Taiwan is a fast, effective, and low-solvent-consumption system for extraction and extensive cleanup [13,39]. The quality assurance/quality control (QA/QC) of this effective extraction and cleanup system meets the criteria in U.S. EPA Method 1613B, 1668A, 1614, 4425, and 4435 as well as the Taiwanese EPA Method [13]. The fast extraction approach coupled with cleanup processes have been applied to various matrices, including soils, sediments, and fish [25,26,40].

To date, no large-scale collections of dioxin-contaminated soil data or national soil dioxin surveillance projects have been carried anywhere in Taiwan, and only a limited number of surveillances or local investigations have been reported [13,25,26,41,42,43]. Many factors need to be considered in large-scale or longitudinal investigations of dioxin-contaminated soil, including the sampling and analytical costs, time needed for the analysis, and the capacity of the instruments used. The DR-CALUX^®^ assay is one of the best methods for the large-scale surveillance of dioxin contaminated soil. In the present study, the DR-CALUX^®^ assay was used to determine the level of soil dioxin contamination in our large-scale national investigation. We further examined whether soil dioxin contamination was associated with soil characteristics (such as brightness, classification into clay, silt, or sand, and moisture level), sampling depth, and sites with different kinds of land use.

## 2. Materials and Methods

### 2.1. Sampling Design

**Figure 1:**
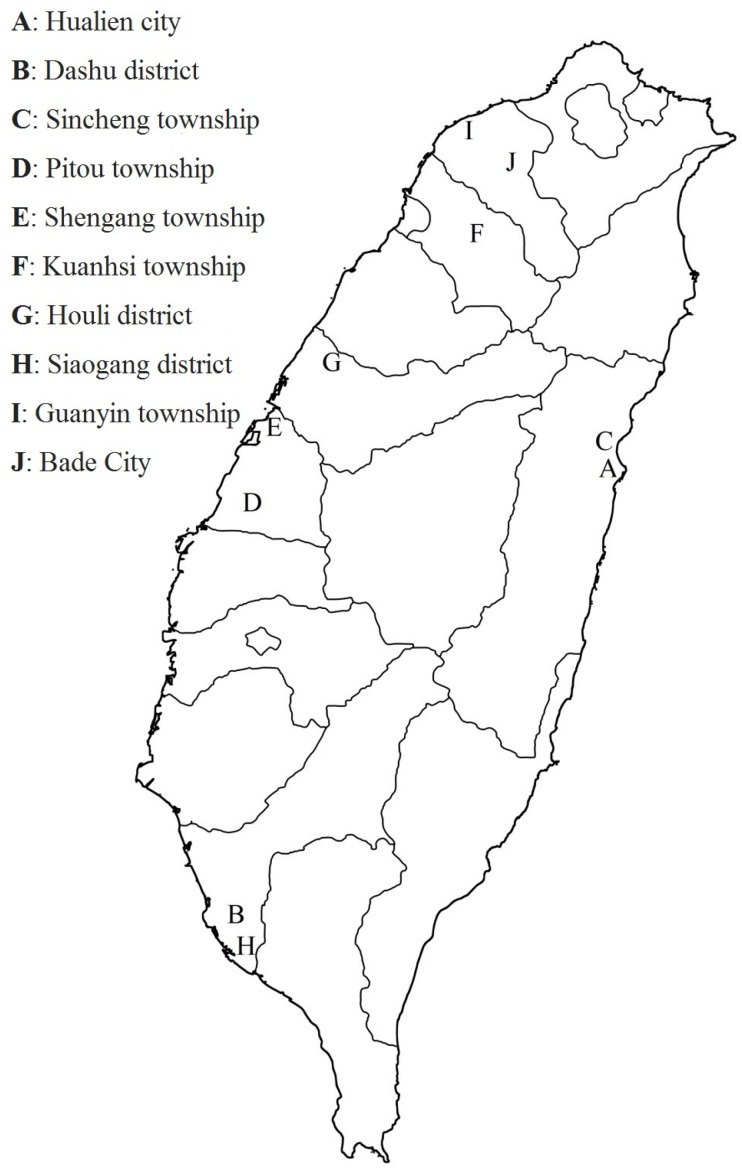
The sampling map of soil dioxin contamination is shown for a national dioxin survey in Taiwan. Soil samples are collected from northern, central, southern, and eastern Taiwan.

This study is part of the first national survey of dioxin contamination in Taiwan from September 2011 to Mar 2013. Purposive sampling was used, and Taiwan was divided into four regions (northern, central, southern, and eastern areas) (Figure 1). 

Owing to the lack of existing data on dioxin contamination in Taiwanese soil, the inventory of dioxin emissions from stationary sources in Taiwan was initially used for the selection of potentially dioxin-contaminated areas and reference sites. Gaussian dispersion models were used to simulate the distribution of dioxin deposition on the soil from stationary points, based on an inventory of dioxin-emission data. The present study used dioxins emission data from the stationary emission sources to simulate dioxins dispersion in each region by the Industrial Source Complex Short-Term Dispersion Model (U.S. EPA Model ISCST3). Source inventory data of dioxins in 2010 were collected from Taiwanese EPA. The emission information of each stationary emission point including the inside diameter of the stack, the height of the stack, emission rate, emission temperature, operation hours per day, and operation days per year was gathered from Department of Air Quality and Noise Control of Taiwanese EPA. The meteorological data in 2010 were purchased from the local weather stations. The grid size of ISCST3 simulation was 50 m × 50 m. After the simulation of various dioxin dispersion models, a number of heavily dioxin-contaminated areas were selected for further examination. In contrast to these areas, a number of the reference sites, with low levels of dioxin contamination, were also selected based on the dispersion simulation. Soil samples were obtained by grid sampling (one sample per five hectares) from the potentially dioxin-contaminated areas and reference sites in each region. Eight hundred samples were initially obtained following this sampling strategy. There was no soil information recorded for 115 of these 800 soil samples, including the outlier values for 14 soil samples. Therefore, only 685 soil samples were considered in the statistical analysis, as the information on these (including soil brightness, sampling depth, soil classification, and soil moisture) was complete. The color of soil was used to describe soil brightness, which varied from light to dark in the order yellow brown, gray, brown, charcoal gray, black brown, and black.

### 2.2. Procedure of Sample Extraction and Cleanup

All solvents used in this work, including toluene, n-hexane, and dimethyl sulfoxide (DMSO), were pesticide residue grade and obtained from Merck (Darmstadt, Germany), Tedia (Fairfield, OH, USA), or Sigma-Aldrich (St. Louis, MO, USA). Standard solutions of 2,3,7,8-tetrachlorodibenzo-*p*-dioxin (2,3,7,8-TCDD) were purchased from Wellington Laboratories (Guelph, ON, Canada). Silica gel (100–200 mesh) was obtained from Fisher (Leicestershire, England, UK). Rapid cleanup was carried out using a CAPE column.

Dry solid samples of 10 g were extracted using an automated Soxhlet system (Extraction System B-811 LSV, BÜCHI, Flawil, Switzerland). Extracted samples were evaporated to near dryness, and then transferred to the CAPE column for cleanup. The cleanup procedure has been described in detail in a number of earlier works [25,39,40]. In brief, the acid-silica column and the CAPE column were prewashed by n-hexane. After Soxhlet extraction, the extract was passed through an acid-silica column. *n*-Hexane (2 mL) was added to the sample extract, and this was then transferred to the column and this procedure was repeated. Ten ml of n-hexane was added to the acid silica column (CAPE column) to elute the sample, and the target analytes were loaded onto the activated carbon mini-column. Duplicate 10-mL portions of *n*-hexane were used to continuously elute all passing solvent containing dioxin-like PCBs from the column. The activated carbon mini-column was removed from the acid silica column and attached to a clean, empty CAPE column. Five mL of a toluene-*n*-hexane (v/v 1:1) mixture was added to the column to elute the remaining dioxin-like PCBs in this fraction, and then this fraction was also discarded. The carbon mini-column was reversed and connected to the empty column, and 20–30 mL of toluene was then added to the column again. All the solvents that passed through column were collected as PCDD/F fractions. Finally, the PCDD/F fractions were evaporated to near dryness, and dissolved in 100 μL of DMSO.

### 2.3. DR-CALUX^®^ Assay of Dioxins

The DR-CALUX^®^ bioassay in the present study followed standard methods, such as NIEA S901.60B (Taiwanese EPA) or USEPA Method 4435. The DR-CALUX^®^ hepatoma cells were purchased from BioDetection Systems B.V. (Amsterdam, The Netherlands). The DR-CALUX^®^ bioassay used a genetically recombinant rat hepatoma (H4IIE) cell line transfected with constructs containing the firefly luciferase reporter gene under the transcriptional control of AhR. The analytical protocols of the DR-CALUX^®^ bioassay have been described in detail elsewhere [44,45]. DR-CALUX^®^ cells were routinely cultured in α MEM supplemented with 10% heat-inactivated fetal calf serum (GIBCO BRL, Gaithersburg, MD, USA) in an incubator (Forma™ Series II 3110 Water-jacketed CO_2_ Incubator, Thermo Scientific, Waltham, MA, USA) with 5% CO_2_ at 37 °C, and cells were seeded in 96-well plates (Catalog no. 167008; NUNC Inc., Naperville, IL, USA) at a density of 1×10^4^ cells/well. One day after plating, the cells were exposed to 2,3,7,8-TCDD standards (0, 0.3, 1, 3, 10, 30, 100, and 300 pM; stock solutions prepared in DMSO) or sample extracts (diluted in culture medium containing 0.8% DMSO (v./v.)) and incubated at 37 °C for 24 h. DR-CALUX^®^ cell monolayers were viewed under an inverted microscope to check for cytotoxic effects after a 24-h incubation of the cells with the extracts at 37 °C. The medium was discarded and the cells were washed with phosphate-buffered saline containing calcium and magnesium (pH 7.4), and lysed with lysis buffer (Catalog no. E1531; Promega, Madison, WI, USA). Luciferase activity was measured using a microplate luminometer (Orion II Luminometer, Berthold Detection Systems, Oak Ridge, TN, USA) and the Luciferase Assay System (Promega), following standard protocols. The luciferase activity was expressed as relative light units (RLUs). RLU values were then transformed into 2,3,7,8-TCDD toxicity equivalents using the BioDetection Excel file. The dioxin levels in DR-CALUX^®^ used in the present study are expressed as bio-analytical equivalents (BEQs) based on previous reports [31,46,47]. The 2,3,7,8-TCDD sigmoid semi-logarithmic dose-response relationship was fitted by the Hill equation (R^2^ > 0.98, *p* < 0.001). BEQ values were required to meet all the QA and QC criteria of bioassay standards in Taiwan (NIEA S901.60B) or U.S. EPA Method 4435.

### 2.4. Statistical Analysis

The Shapiro-Wilk test showed that the distribution of dioxin-BEQ levels from the 800 soil samples was not normal. Nonparametric analysis methods including chi-square, Fisher’s exact, Mann-Whitney *U*, and Kruskal-Wallis H tests were used to examine the mean differences among the variables. Logistic regression models were used to examine the correlations between high and low dioxin-BEQ levels and the different soil characteristics (e.g., soil brightness). Logarithmically transformed dioxin-BEQs values were used in a linear regression model to determine their relationships. Two-way analysis of variance (ANOVA) was performed to determine whether the interactions and differences in the dioxin-BEQs are significantly related to different soil characteristics. The significance level in the present study was 0.05. The statistical computations in this study were performed using the Statistical Product and Service Solutions (SPSS) software, version 12.0.

## 3. Results

The dioxin-BEQ level in 800 samples of Taiwanese soil was 36.0 ± 56.7 (mean ± standard deviation (SD)) pg-BEQ/g dry weight (d.w.) and not significantly different in comparison with that in the 685 soil samples (30.8 ± 40.2 pg-BEQ/g d.w.). Table 1 shows the descriptive statistics for the 685 soil samples and their dioxin contamination values (dioxin-BEQs), various details of the soil characteristics, including sampling location and depth, soil classification, soil moisture, soil brightness, land use, and dioxin contamination levels. Related to dioxin values by sampling location in Taiwan, dioxin levels by sampling depth (*p* = 0.036) and land use (*p* < 0.001) were shown to differ significantly by chi-square or Fisher’s exact test analysis. Furthermore, statistical analysis of the soil dioxin data using the nonparametric Kruskal-Wallis H method also showed regional differences in levels. The mean dioxin-BEQs in the soil samples from eastern Taiwan (mean ± SD: 7.80 ± 5.08 pg-BEQ/g d.w.) was significantly lower than those from western Taiwan, such as northern (61.8 ± 62.3 pg-BEQ/g d.w.), central (22.2 ± 12.8 pg-BEQ/g d.w.), and southern (24.9 ± 26.3 pg-BEQ/g d.w.) regions. A high linear correlation was found between dioxin WHO_2005_-TEQs or I-TEQ and dioxin-BEQs in 25 soil samples randomly selected from the 800 samples (WHO_2005_: adjusted R^2^ = 0.857, *p* < 0.001; I-TEQ: adjusted R^2^ = 0.893, *p* < 0.001). The mean ratio of dioxin values obtained with DR-CALUX^®^ *vs.* HRGC/HRMS was 4.55 (SD = 2.15) for WHO_2005_ TEQs and 3.83 (SD = 1.40) for I-TEQs, respectively. 

**Table 1:** Descriptive analysis of a dioxin survey in Taiwanese soil (N = 685).

Soil Characteristics		Location in Taiwan				*X*^2^ (*p*) ^a^
		Northern (n = 184)	Central (n = 247)	Southern (n = 132)	Eastern (n = 122)	
		Frequency (Number)				
**Soil brightness**						0.696
Light						
Yellow brown		7	9	8	2	
Medium						
Gray		31	39	18	18	
Brown		94	143	72	58	
Charcoal gray		43	47	28	39	
Dark						
Black brown		7	8	5	3	
Black		2	1	1	2	
**Sampling depth**						0.036 *
10 cm		128	168	107	82	
15 cm		56	79	25	40	
**Soil classification**						0.321
Clay		4	5	6	1	
Silt		2	4	5	2	
Sand		178	238	121	119	
**Soil moisture**						0.794
Wet		70	103	53	45	
Dry		114	144	79	77	
**Land use**						<0.001 ***
Wasteland		42	39	6	57	
Industry		44	23	88	43	
Park near residential area		10	20	13	15	
Food production near industrial area **^b^**		84	158	0	4	
Others **^c^**		4	7	2	3	
Missing **^d^**		0	0	23	0	
**Dioxin concentration**		Mean ± SD (pg-BEQ/g d.w.)				*p* **^e^**
DR-CALUX^®^ assay		61.8 ± 62.3	22.2 ± 12.8	24.9 ± 26.3	7.80 ± 5.08	<0.001 ***

Notes: **^a^** Fisher’s exact test was calculated when the expected values in any of the cells of a contingency table were below 5, and chi-square test was used when they were above 5. **^b^** Food production district: agriculture, fisheries, and livestock. **^c^** Schools, public buildings, and government institutions. **^d^** No coding or missing data. **^e^** Kruskal-Wallis H test. * *p* < 0.05, *** *p* < 0.001.

**Figure 2:**
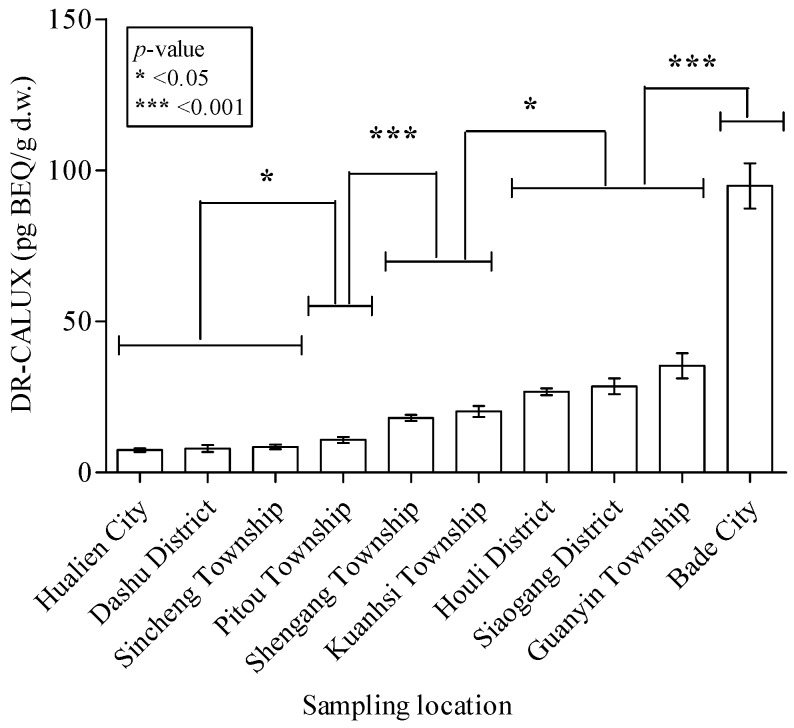
Dioxin concentrations in Taiwanese soil from 10 cities, districts, or townships (n = 685). The dioxin levels are markedly higher in soil collected from Bade City than in soil collected from the other surveyed areas (*p* < 0.001); significantly higher in Guanyin, Siaogang, and Houli than in the other areas except for Bade City (*p* < 0.05 or *p* < 0.001); significantly higher in Kuanhsi and Shengang than in Pitou, Sincheng, Dashu, and Hualien City (*p* < 0.001); slightly higher in Pitou than in Sincheng, Dashu, and Hualien City (*p* < 0.05), and lowest in Sincheng, Dashu, and Hualien City among the 10 selected survey areas (*p* < 0.05 or *p* < 0.001).

The average soil dioxin-BEQ level in 10 subareas of the four regions is shown in Figure 2. The highest mean soil dioxin-BEQ level was in Bade City (94.9 ± 7.50 pg-BEQ/g d.w.) and was 2- to 20-fold greater in magnitude than in the other areas (*p* < 0.001). The BEQ level in soil from Guanyin (35.3 ± 4.17 pg-BEQ/g d.w.), Siaogang (28.5 ± 2.63 pg-BEQ/g d.w.), and Houli (26.7 ± 1.14 pg-BEQ/g d.w.) were significantly higher than those from the all other areas examined in this work, except for Bade City (*p* < 0.05 or *p* < 0.001). Dioxin-contaminated soil samples from Kuanhsi (20.2 ± 1.81 pg-BEQ/g d.w.) and Shengang (18.0 ± 1.05 pg-BEQ/g d.w.) have significantly higher dioxin-BEQ levels than those from Pitou (10.8 ± 1.02 pg-BEQ/g d.w.), Sincheng (8.43 ± 0.742 pg-BEQ/g d.w.), Dashu (7.91 ± 1.16 pg-BEQ/g d.w.), and Hualien (7.37 ± 0.584 pg-BEQ/g d.w.) (*p* < 0.001). The mean dioxin-BEQ levels in soil from Sincheng, Dashu, and Hualien in eastern Taiwan were the lowest among all surveyed areas, including Pitou, which has the lowest soil dioxin-BEQ level among the sampling areas in western Taiwan.

**Figure 3:**
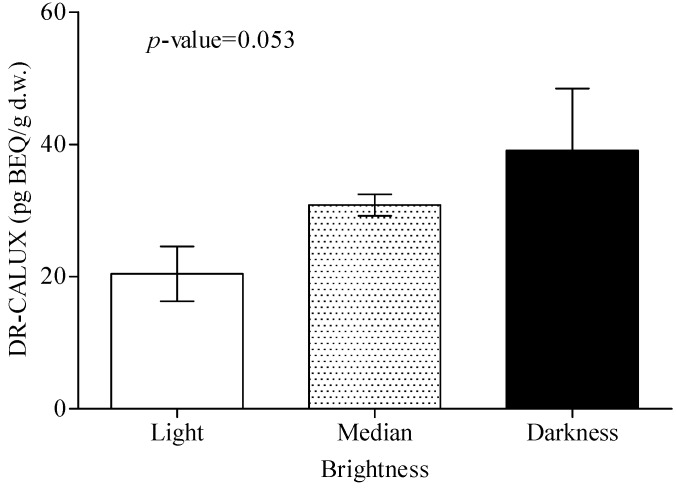
Dioxin concentrations are shown for soils of different brightness (three colors) (n = 685). Dioxin levels are similar in the three soils of different color and marginally significantly different between light and dark soils (*p* = 0.053).

**Figure 4:**
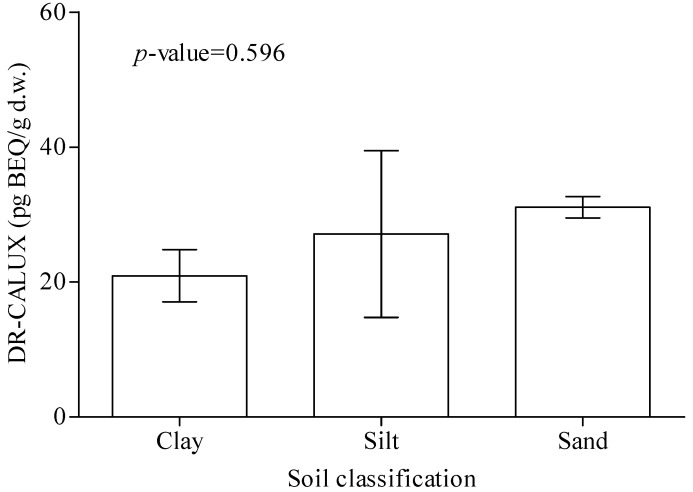
Dioxin-BEQs in clay, silt, and sand in our soil samples (n = 685). Dioxin concentrations are similar among clays, silts, and sands (*p* = 0.596).

**Figure 5:**
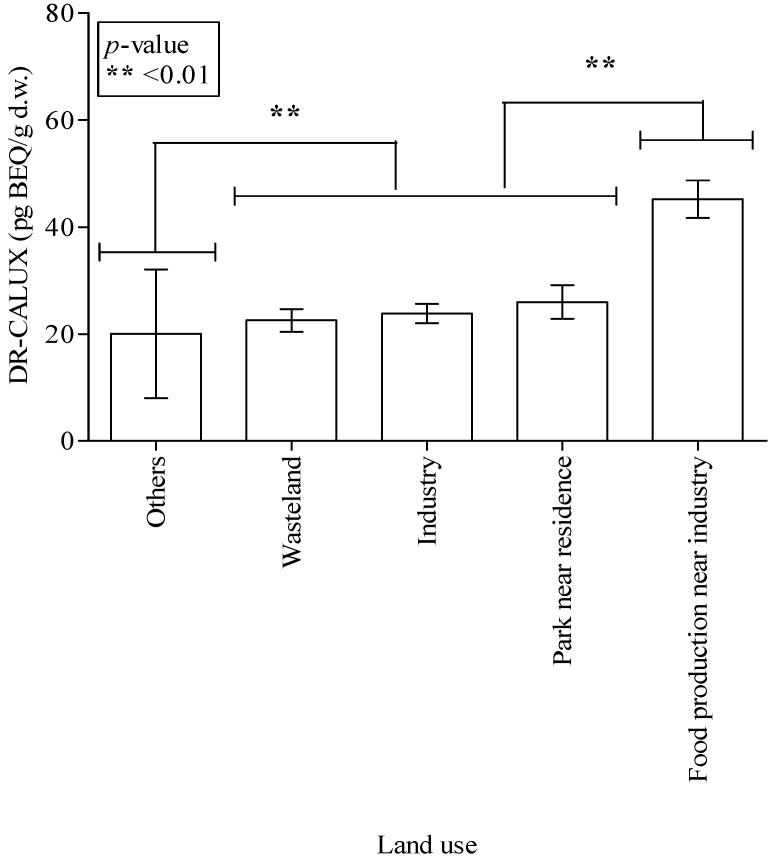
Dioxin concentrations in Taiwanese soil depend on land use (n = 685). The dioxin concentration in soil used for food production near an industrial area is notably higher than that in soil from the other areas (*p* < 0.01 or *p* < 0.001). Soil dioxin levels in parks near residential areas, industrial areas, and wasteland areas are significantly higher than those in land used for other purposes (*p* < 0.01).

Data analysis to determine whether various soil characteristics are associated with dioxin contamination (Figure 3 and Figure 4) showed no significant differences in dioxin-BEQ level between soil samples with different brightness (*p* = 0.053) or classification (*p* = 0.596). There were also no significant differences in dioxin-BEQ level between samples from different sampling depths (data not shown). With regard to land use, notably soil from food production areas near an industrial area had the highest dioxin-BEQ level (45.2 ± 3.49 pg-BEQ/g d.w.; *p* < 0.01 or *p* < 0.001) (Figure 5). Soil samples from industrial areas (23.8 ± 1.80 pg-BEQ/g d.w.), wasteland (22.5 ± 2.13 pg-BEQ/g d.w.), and parks near residential areas (26.0 ± 3.16 pg-BEQ/g d.w.) showed similar levels of dioxins contamination, with significantly higher levels compared to those from land used for other purposes (20.0 ± 12.0 pg-BEQ/g d.w.) (*p* < 0.01).

An univariate logistic regression model was used to examine the odds ratios (ORs) of soil dioxin-BEQs > the third quartile (33.4 pg-BEQ/g d.w.) for soil characteristics compared to the controls (dioxin-BEQs ≤ 3rd quartile) (Table 2 (n = 662)). The ORs of 2.43, 1.47, and 2.83 for soil sampling location, land use, and soil brightness, respectively, were significantly higher than those for the controls. Furthermore, a stepwise multiple linear regression model revealed that the dioxin-BEQ levels in 662 soil samples were significantly predicted by four variables (soil sampling location, soil brightness, land use, and soil moisture) (Table 3). A two-way ANOVA test used to examine the main components of dioxin contamination in the present study failed to show any interaction effects between dioxin-BEQ level and soil characteristics (data not shown). The log_10_-transformed soil dioxin-BEQ levels were well explained using the parameters of soil location (*β* = 0.097, *p* < 0.001), land use (*β* = 0.065, *p* < 0.001), soil brightness (*β* = 0.170, *p* < 0.001), and soil moisture (*β* = 0.051, *p =* 0.020), with the adjusted R^2^ of 0.947 (*p* < 0.001).

**Table 2:** Univariate logistic regression to obtain odds ratios of Taiwanese soil dioxin concentrations >3rd quartile (33.4 pg-BEQ/g d.w.) according to soil sampling location, land use, soil brightness, soil moisture, and soil depth (n = 662).

DR-CALUX (pg-BEQ/g d.w.)	Odds Ratio				
	Soil Sampling Location	Purpose of Land Use	Soil Brightness	Soil Moisture	Soil Depth
<33.4 **^a^**	1.00	1.00	1.00	1.00	1.00
>33.4	2.43	1.47	2.83	1.14	0.961
*p*-value	<0.001 ***	<0.001 ***	0.009 **	0.574	0.835

Notes:^** a**^ The 75th percentile (3rd quartile) of DR-CALUX concentration was 33.4 pg BEQ/g d.w., ** *p* < 0.01, *** *p* < 0.001.

**Table 3:** Stepwise multiple linear regression analysis identifying the soil characteristics predicting DR-CALUX concentration (n=662).

Dependent Variable	Predictors	Beta	*p*-value	Adjusted R Square	*p*-value
Log_10_ DR-CALUX (pg-BEQ/g d.w.)	Soil sampling location	0.097	<0.001 ***	0.947	<0.001 ***
	Purpose of land use	0.065	<0.001 ***		
	Soil brightness	0.170	<0.001 ***		
	Soil moisture	0.051	0.020 *		

Notes: * *p* < 0.05, *** *p* < 0.001.

## 4. Discussion

This study was the first national survey to use bioassay for determining dioxin levels in Taiwanese soils (n = 800). With regard to the use of AhR reporter gene bioassays to investigate dioxin-contaminated soils, the mean value in the present study was higher than those from the other studies [8,28,29]. Dioxin contamination levels in eastern Taiwan ranged from 1.3 to 45.6 pg BEQ/g d.w. (mean = 7.8 pg-BEQ/g d.w., SD = 5.08 pg-BEQ/g d.w.), and thus were slightly higher than those in farmland soil near villages located in northwestern Mexico (4.2 pg-BEQ/g d.w) [28] and soil near the Bohai Sea in China (3.8 pg-BEQ/g d.w.) [29]. Our values for the soil from eastern Taiwan were comparable to those for soils around waste incineration plants located at Zhejiang province in southeastern China (11.0 pg-BEQ/g) [8], and soil near the Yellow Sea on the western coast of South Korea (9.2 pg BEQ/g d.w.) [29]. The present study also showed a strong linear correlation between the dioxin levels obtained via the HRGC/HRMS and DR-CALUX^®^. A Chinese study also found a similar correlation between the results of a chemical assay and bioassay (R = 0.87) [8], while several reports found a nonlinear relationship between the results of an *in vitro* bioassay and the results of the HRGC/HRMS method in dioxin-contaminated soils or sediments [25,27,32,48]. Not all the reports of AhR reporter gene assays including CALUX bioassay found a linear relationship between BEQs and TEQs. For instance, it seems to be hardly applicable to the monitoring of dioxins in the food chain, taking into account the fact that congener patterns change from one sample to another. DR-CALUX values are overestimated compared with TEQ values from HRGC/HRMS analysis. Although the fraction of PCDD/Fs in the final extract had been successfully separated from the other fractions, which were possibly contained in certain AhR-ligand compounds, after the CAPE column cleanup, the PCDD/Fs fraction still existed certain PCDD/Fs and residual AhR activated compounds to induce DR-CALUX activation except for 2,3,7,8-substituted PCDD/Fs. Considering the background levels of dioxins contamination in Taiwanese soils, Jou and his research team investigated background levels of soil dioxin-TEQs in the agriculture and natural preserve areas of Taiwan between 2001 and 2002 [43]. Mean levels of soil dioxin-TEQs were 3.37 and 2.20 pg I-TEQ/g d.w. in the agriculture farms (n = 96) and natural preserve areas (n = 11), respectively [43]. If we used the ratio of BEQs/I-TEQs as 3.83 to estimate soil I-TEQ levels in the present study, soil dioxin-TEQ levels were presented as 2.82 pg I-TEQ/g d.w. in Pitou (10.8 pg-BEQ/g d.w.), 2.20 pg I-TEQ/g d.w. in Sincheng (8.43 pg-BEQ/g d.w.), 2.07 pg I-TEQ/g d.w. in Dashu (7.91 pg-BEQ/g d.w.), and 1.92 pg I-TEQ/g d.w. in Hualin (7.37 pg-BEQ/g d.w.) with the similarity of background levels of soil dioxin-TEQs reported in the Jou’s study [43]. Soil dioxin levels in Pitou, Sincheng, Dashu, and Hualin might be approximately close to dioxin background levels in Taiwanese soil based on our results.

Based on the results of multiple linear and univariate logistic regression models (Table 2 and Table 3), the key soil characteristics affecting dioxin contamination of the 662 soil samples were the three variables of sampling location, land use, and soil brightness. The sampling location was the most statistically significant of the three key soil variables in these two regression models based on our findings. The Spearman rho correlation coefficient of soil sampling location and land use was slightly significant (R = 0.141, *p* = 0.046). In Figure 5, significant differences in dioxin-BEQs were found among different land use purposes, and dioxin-BEQs were especially high in soil used for food production near industrial areas. Soil dioxin-BEQs were not higher in industrial areas than in food production areas near industrial areas. A previous dioxin survey of coastal soil near the Yellow Sea in China and South Korea [49] found that soil dioxin levels were significantly higher in industrial areas than in municipal, agricultural, and other areas. 

The distribution of dioxin contamination differed between sites and areas in the present study (Table 1 and Figure 2). Dioxin-BEQ levels differed significantly between regions or subareas, such as northern, central, and southern Taiwan but not between potentially dioxin-contaminated areas and reference sites throughout Taiwan. Initially, this study used the dispersion model (ISCST3 model) to select potentially dioxin-contaminated areas and reference sites in each region, and then collected samples for DR-CALUX^®^ bioassay of soil dioxins. According to the results of dioxin surveillances in the present study, soil dioxin-BEQs (20.2 pg-BEQ/g d.w.) in Kuanhsi, which was chosen as the selected reference site in northern Taiwan, did not listed in the group of the background-level soil dioxins in Taiwan. Sincheng and Hualin were recognized as the potentially dioxin-contaminated area and reference site, respectively, in the eastern Taiwan to present the non-significant difference of soil dioxin-BEQs. Compared with the values from dioxins dispersion simulation, the elevated or highest soil dioxin concentrations analyzed by DR-CALUX^®^ bioassay among dioxin measurements were usually inconsistent in most selected sampling sites. Furthermore, dispersion is not the same than deposition, which is extremely influenced by wet processes like rain wash. Anthropogenic pollution (for an example, illegal waste disposal) also influenced dioxins contamination in soils except for airborne deposition. The results of the present study do not entirely support our initial hypothesis that the dioxin dispersion model can be to used determine the appropriate sampling areas. The results suggest that the model underestimated dioxin contamination in northern Taiwan and overestimated it in eastern Taiwan. A recent study, which estimated global deposition of airborne dioxins, predicted that most of the annual dioxin production (*i.e.*, 163 kg-TEQ (or 57%)) would settle onto land areas. Estimates were based on a complicated set of assumptions, including gross domestic product, annual dioxin production in each country, air advection and diffusion, grasshopper effects, temperature, geographic information, and wind speed and direction [50]. Vassura *et al.* (2011) found that patterns of dioxin distribution in the stack emissions of an incineration plant differed from surrounding soil, thus revealing that soil dioxin contamination may not be associated with dioxin atmospheric deposition fluxes from the incinerator. Although some sampling bias may have occurred in the present study, a number of significant spatial differences in the dioxin distribution were found. A large survey of the coastal soil near the Bohai Sea in northeastern China and near the Yellow Sea in western South Korea using an AhR *in vitro* bioassay revealed that the levels of dioxin-like compounds or AhR agonists in soils differed among locations and between countries [29]. Several studies using chemical assays reported spatial effects on the distribution of dioxins in soils [23,49]. Naile *et al.* used the HRGC/HRMS approach to assay the same soil samples assayed by Hong *et al.*, and found that similar results were obtained with the *in vitro* bioassay [49]. In a large-scale study in China that investigated soil dioxins in high mountain areas extending from Wolong to the eastern Tibet-Qinghai Plateau [23], dioxin contamination was positively associated with altitude, probably due to long-range atmospheric transport and subsequent aerial deposition, although the dioxin levels were extremely low.

Although the mean value of dioxin-BEQs in sand was higher than those in clay and silt, but their differences were not significant (p = 0.596) (Figure 5). Our result was different from the report in a Canadian research that found the increase order of soil dioxin-TEQ levels to be followed as sand, clay, and the organic soil [51]. They also indicated that vertical migration of PCDD/Fs in organic soils, clay, and sand distinctly differed depending on the three types of soil [51]. In the present study, soil brightness had marginally significant association with soil dioxins contamination (*p* = 0.053) (Figure 3). Logistic or linear regression analysis identified soil brightness as a significant variable predicting soil dioxins contamination in Taiwan (Table 2 and Table 3). Soil dioxins contamination may be related to soil brightness particularly to dark soil which may be more contaminated with dioxins than light colored soil. We could not find the direct evidence in the published literatures showing the associations between soil color and dioxin contents of soils to support our finding. It is well known that the general effect of organic matter is the darkening of the soil. Soil organic matter (SOM) is composed of amorphous organic matter (AOM) and carbonaceous materials such as black carbon (BC), coal and kerogen. Black carbon is as a fraction of total organic carbon. Several reports indicated that higher organic matter was associated with elevated soil dioxin levels or enhancement of AhR mediated activation [52,53,54,55]. Black carbon is as a fraction of total organic carbon. PCDD/Fs are significantly correlated with black carbon (R = 0.34, *p* < 0.01) and total organic carbon (R = 0.31, *p* < 0.05) [56]. The findings in the present study indicate that soil brightness may affect dioxins contamination in soil, but leave unanswered the question of why this characteristic may be correlated with the distribution of dioxins in soil. Statistical analysis using the Mann-Whitney U, Kruskal-Wallis H, univariate logistic and multiple linear regression tests in the present study identified sampling location, land use, and soil brightness as significant variables correlated with dioxins contamination in selected Taiwanese soil samples. This study is valuable because it is the first to examine whether soil characteristics are correlated with dioxin contamination in soil and the first to investigate large-scale dioxin contamination in Taiwanese soil. The results show that DR-CALUX^®^ assay is a powerful, useful, and high throughput tool that can be used to detect dioxin levels in a large number of soil samples.

## 5. Conclusions

This study examined dioxin levels in 800 soil samples from Taiwan. The DR-CALUX^®^ bioassay coupled with combination of the automated Soxhlet system and the fast cleanup column, namely the CAPE column, is an excellently fast-screening tool to investigate soil dioxin contamination on a large scale. According to our findings, dioxins distribution in Taiwanese soils was mainly influenced by the three soil characteristics including location, land use, and soil brightness. Although soil brightness is probably correlated with dioxins contamination in the present study, the current evidence still does not justify this conclusion because the question of why soil brightness affects soil dioxins contamination has not been answered. Thus, future studies are encouraged and needed to confirm this.
